# Knowledge, attitudes, and practices on child and adolescent mental health among healthcare workers in sub-Saharan Africa: a scoping review

**DOI:** 10.1186/s13033-024-00644-8

**Published:** 2024-07-16

**Authors:** Beatrice Mkubwa, Vibian Angwenyi, Brenda Nzioka, Charles R. Newton, Marit Sijbrandij, Amina Abubakar

**Affiliations:** 1 Institute for Human Development, Aga Khan University, Nairobi, Kenya; 2 Department of Clinical, Neuro- and Developmental Psychology, WHO Collaborating Center for Research and Dissemination of Psychological Interventions, Amsterdam Public Health Research Institute, Vrije Universiteit Amsterdam, Amsterdam, The Netherlands; 3 Neuroscience Unit, KEMRI-Wellcome Trust, Center for Geographic Medicine Research Coast, Kilifi, Kenya; 4 Department of Psychiatry, University of Oxford, Oxford, UK

**Keywords:** Knowledge, Attitudes, Practices, Child and adolescent mental health, Healthcare workers, Sub-Saharan Africa

## Abstract

**Introduction:**

Child and adolescent mental health is a global priority. In sub-Saharan Africa, despite the high burden, there is a gap in health services for children and adolescents with mental health disorders. To bridge this gap, healthcare workers require a good understanding of child and adolescent mental health, the right attitude, and practices geared to improving child and adolescent mental health. This scoping review examined the knowledge, attitudes, and practices related to child and adolescent mental health among sub-Saharan African healthcare workers.

**Methods:**

The search was restricted between January 2010, the year when the Mental Health Gap Action Programme guidelines were launched, and April 2024. The review followed the methodological framework proposed by Arksey and O’Malley for conducting scoping reviews. The databases searched included CINHAL, PubMed, Web of Science, PsycINFO, and grey literature databases. Additional articles were identified through cited references of the studies included. A data extraction template was used to retrieve relevant text. A narrative synthesis approach was adopted to explore the relationships within and between the included studies.

**Results:**

The literature search yielded 4658 studies. Among these, 817 were identified as duplicates, and 3740 were excluded after screening. Only twenty-one articles met the criteria for inclusion in the review. The findings showed that healthcare workers have insufficient knowledge of child and adolescent mental health, hold negative attitudes toward children and adolescents with mental health problems, and exhibit poor practices related to child and adolescent mental health.

**Conclusion:**

It is crucial to build capacity and improve healthcare workers’ practices, knowledge, and attitudes toward child and adolescent mental health in sub-Saharan Africa. This could lead to better access to mental health services for children and adolescents in the region.

**Supplementary Information:**

The online version contains supplementary material available at 10.1186/s13033-024-00644-8.

## Introduction

Globally, there is a high burden of Child and Adolescent Mental Health (CAMH) disorders [1–3]. The prevalence of CAMH disorders is estimated to be between 10 and 20%, with one in five children experiencing mental health problems [4, 5]. CAMH disorders are a leading cause of health-related disability in children and adolescents, and their effects can persist throughout life [6]. The Mental Health Gap Action Programme (mhGAP) Intervention Guide classifies CAMH disorders into developmental disorders such as autism, emotional disorders such as adolescent depression, and behavioral disorders such as conduct disorders [7].

In sub-Saharan Africa (SSA), one in every seven children and adolescents (14.3%) has a serious psychological problem [8]. A systematic review and meta-analysis of SSA countries, which included 46, 464 adolescents from 22 studies, reported a prevalence of mental health disorders was 23% [9]. Another systematic review of 97,616 adolescents found the following prevalence estimates: 40.8% for emotional and behavioral difficulties, 29.8% for anxiety disorders, 26.9% for depression, 21.5% for PTSD, and 20.8% for suicide ideation [10]. A significant variation from the prevalence reported in a review of 14 studies from 11 high-income countries, including 61, 545 children aged 4 to 18, that reported the prevalence of anxiety to be 5.2%, attention-deficit hyperactivity 3.7%, conduct 1.3%, and depressive disorders 1.3% [11]. The high burden of CAMH disorders is compounded by a lack of health services and in many SSA countries, the treatment gap can be as high as 75% [8, 9]. For instance, in Kenya, there are fewer than 500 specialist mental health workers serving a population of over 50 million [12].

CAMH care in SSA countries is influenced by a complex set of factors, including unique contexts and challenges. Factors such as poverty, limited access to mental healthcare, and inadequate healthcare infrastructure pose significant obstacles in mental healthcare in already overstretched health systems [13]. Due to competing health and development priorities and insufficient funds, mental healthcare in many SSA countries is severely underfunded and often under-prioritized [14]. Data collection systems often overlook mental health, contributing to policymakers’ lack of understanding of the extent of the problem [15]. In most SSA countries, the CAMH services are mostly available at tertiary health facilities [16–18]. Additionally, there are no clear referral pathways [19]. The integration of services in primary healthcare is also suboptimal [20]. Cultural beliefs and stigma surrounding mental illness often lead to neglect of mental health needs [21]. The mental health needs of children and adolescents with these disorders are frequently overlooked, and early identification is limited [22].

Recommendations have been made to decentralize CAMH care services to primary healthcare facilities to establish a robust health system for CAMH services and build capacity even at the lowest level of health systems [23–25]. However, for this process to be effective and successful, it is crucial to enhance the knowledge, attitudes, and practices (KAP) of healthcare workers (HCWs) regarding CAMH [26–30]. In most SSA countries, HCWs can fall into several cadres but mostly include nurses, midwives, medical doctors, clinical officers, psychologists, social workers, and community health workers [12]. Despite the many cadres, only a few HCWs within these cadres, such as psychiatrists, psychologists, and mental health nurses are adequately trained to provide mental healthcare [31]. Furthermore, an even smaller proportion of these professionals have specialized in CAMH. The few who possess such training are employed in higher-level healthcare facilities and the private sector [32]. This translates to inadequate knowledge about CAMH and subsequent poor attitude and practices among this workforce [14]. HCWs’ knowledge of CAMH can be described as understanding CAMH concepts, the different CAMH disorders, their symptoms, causes, diagnostic criteria, and available treatment options [26, 27, 30]. Attitudes refer to the beliefs, opinions, and emotional responses of HCWs toward individuals with CAMH disorders [27, 33]. Positive attitudes entail empathy, carefulness, and a non-judgmental approach that recognizes the importance of mental health in children and adolescents [29, 34]. Healthcare workers’ practices involve assessing, treating, and managing CAMH disorders [28, 35, 36]. Therefore, by enhancing the KAP of HCWs regarding CAMH, it becomes feasible to establish a robust health system for CAMH services and build capacity even at the lowest level of health systems, facilitating the decentralization of CAMH care services to primary healthcare facilities.

In 2008, the World Health Organization (WHO) released the mhGAP Intervention Guide, which was updated to version 2 in 2016 [7, 37]. Recently, in November 2023, the WHO updated the mhGAP based on research evidence [38]. These guidelines are used to assess and diagnose mental health issues, including those in children and adolescents, in non-specialist healthcare settings, such as primary healthcare facilities [7, 37, 38]. The release of the mhGAP intervention guide was a significant milestone in integrating mental health into primary healthcare systems and building HCWs’ capacity to provide mental health services, including CAMH services.

In SSA, studies on mental health, including CAMH, have received increased attention since the launch of the mhGAP guidelines. Several studies have reported on the KAP of HCWs regarding CAMH-related issues in SSA. For example, a study conducted in Nigeria found that non-specialized medical doctors had limited knowledge of autism, an important CAMH disorder [39]. Similarly, in Uganda, HCWs had difficulties in making a diagnosis, had a poor understanding of autism symptoms, and had misconceptions about how autism presents [40]. Additionally, children and adolescents with mental illness often experience stigma from HCWs [27, 41], highlighting the need for capacity building programs aimed at improving HCWs’ KAP toward CAMH [26, 40, 42]. Currently, some countries in sub-Saharan Africa, such as Nigeria, are implementing formal CAMH training programs to build human resource capacity for CAMH needs [43]. Following the launch and integration of mhGAP, it is important to summarize the state of evidence on the KAP on CAMH among HCWs from SSA given that the guidelines were launched to bridge the gap in HCWs’ KAP on mental health, including CAMH, especially in non-specialist healthcare settings. Until now, to our knowledge, no scoping or systematic reviews on this topic have been conducted on this topic. This review aims to fill this gap.

This scoping review aimed to map out research evidence on knowledge, attitudes, and practices regarding CAMH among HCWs in SSA. The findings of this review could aid in the development of policies and interventions tailored to bridge the gap on KAP about CAMH among health HCWs in this context.

## Methods

The scoping review was guided by the methodological framework proposed by Arksey and O’Malley for conducting a scoping review [44]. Arksey and O’Malley’s framework comprises several steps: first, formulating the research question; second, identifying relevant studies; third, conducting the study selection and data charting; thereafter, consolidating, summarizing, and presenting the results; and finally, an optional step involving a consultation exercise [44, 45]. The final review protocol was registered prospectively with the Open Science Framework on 19th February 2023. The protocol can be accessed using this link: https://osf.io/rv92q/.

### Eligibility criteria

To be included in the review, the study population needed to be HCWs, such as clinicians, nurses, doctors, community HCWs/volunteers, psychologists, or psychiatrists. The articles were included if they examined the knowledge, attitudes, or practices toward CAMH, CAMH disorders, child mental health, or adolescent mental health, as reported by the studies.

Any original empirical research, such as randomized controlled trials, quasi-experimental designs, pre-post evaluations, open trials, qualitative studies, quantitative studies, and mixed-method studies, were considered for inclusion. Articles published in any language were considered. The time for inclusion was from 1st January 2010 to 06th April 2024, which includes more than a decade of research regarding HCWs’ KAP since the introduction of the mhGAP in 2008. Reviewing literature from 2010 provides a 2-year window of opportunity for implementing the mhGAP guidelines since its launch. The KAP framework was adopted for use in this review, over others such as the Mental Health Literacy framework [46], given its holistic approach to comprehensively assess our topic, identify gaps, establish baseline assessments, and facilitate cross-cultural comparisons, including in mental health [47–51]. The KAP framework is widely used for demonstrating societal context in public health research [52]. The information generated through studies utilizing the KAP framework can be used to develop strategies, including mental health-related, with a focus on improving the behavioral and attitudinal changes driven by the level of knowledge, perceptions, and practices [47, 48, 50]. The articles included reported findings from SSA countries, which are listed in Appendix 1 of Additional file 1.

### Information sources

An initial basic search was conducted in the PubMed database to identify relevant sources of evidence by refining key concepts such as “child and adolescent mental health”, “knowledge”, “attitudes”, “experiences”, “practices”, and “healthcare professionals.” The text words found in the titles and abstracts of pertinent articles, as well as the index terms used to describe the articles, were used to develop a comprehensive search strategy for the review. The refined search strategy concepts and their synonyms were connected using Boolean operators “OR” and “AND” and then applied to all the databases listed, including CINHAL, PubMed, Web of Science, and PsycINFO. Furthermore, gray literature databases and cited references of included studies were searched, such as Think Tank Search and Open Grey. The final search strategy used in PubMed can be found in Appendix 2 of Additional file 1. Additionally, reference tracking and hand-searching were conducted to identify any relevant articles published after the indexing process.

### Selection of sources of evidence

Following the search, all identified citations were collected and uploaded into the Evidence for Policy and Practice Information (EPPI) Reviewer, a software used to conduct literature reviews [53]. Duplicates were removed, and titles and abstracts were screened against the review’s inclusion criteria. Relevant sources of evidence were retrieved in full, and their citation details were imported into EndNote X9. The full text of selected citations was thoroughly assessed against the inclusion criteria by two independent reviewers, and reasons for excluding sources of evidence that did not meet the criteria were recorded and reported. Any disagreements between the reviewers were resolved through discussions with additional reviewers in the authorship.

### Data charting process

Data from the articles included in the review were extracted and charted by two independent reviewers (BM and BN) using a data extraction template designed in MS Excel. The reviewers filled in the template independently (see Additional file 1: Appendix 3). The data extracted were on the study population, concept, context, study methods, and key findings that were relevant to the review question. The data extraction tool was modified and revised as necessary while extracting data from each article included. Any disagreements between the reviewers were resolved through discussions or consultation with an additional reviewer.

### Data items

We extracted information regarding various aspects of the articles, such as their country of origin, study methodology, intervention features (if present), and HCWs’ attitudes, knowledge, or practices regarding child or adolescent mental health.

### Quality assessment of included papers

The review assessed the potential bias of the articles included by employing the Newcastle–Ottawa Scale [54]. This scale evaluated studies across three domains: participant selection, comparability of study groups, and ascertainment of exposure or outcome. Cohort studies were awarded a maximum of nine stars, while cross-sectional studies could receive up to ten. The final star rating was then used to categorize studies as unsatisfactory (0–4), satisfactory (5–6), good (7–8), or very good (9–10) [54]. Additional information can be found in the supplementary file.

### Data analysis and presentation

Data were extracted and analyzed in MS Excel^©^. Thematic analysis was used to give a narrative account of the data extracted from the studies included [55]. Data were extracted around the following outcomes: knowledge of CAMH, attitudes toward children and adolescents with mental health problems, perceptions toward CAMH problems, and management practices of CAMH problems. A narrative summary accompanied by tabulated results was used to present the results related to the review objective.

## Results

**Fig. 1:**
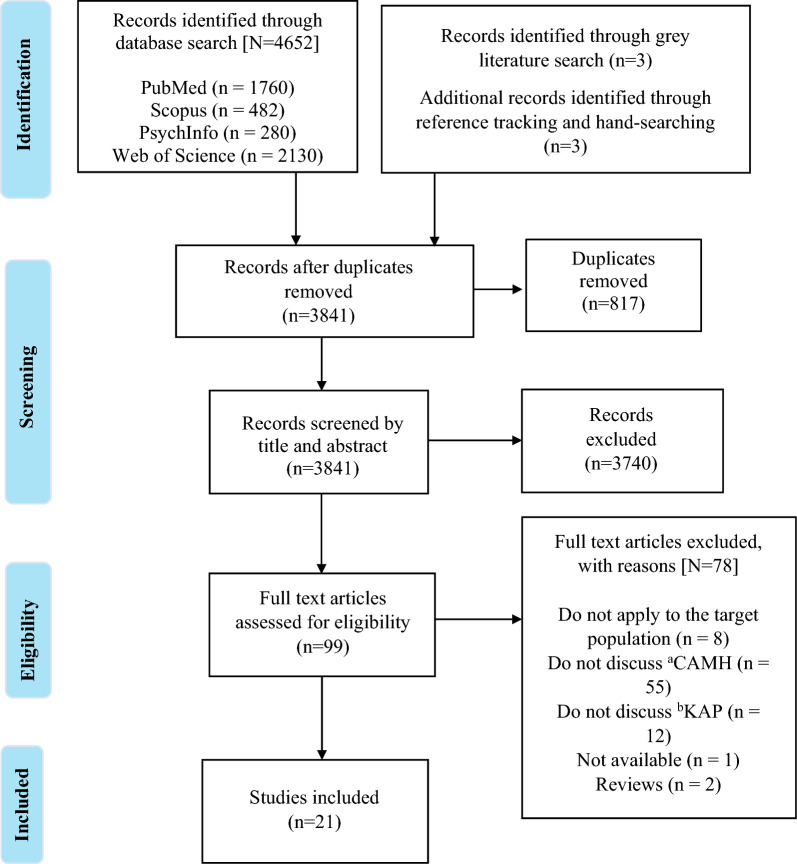
Flow chart diagram showing the scoping review process. ^a^CAMH: child and adolescent mental health; ^b^KAP: knowledge, attitudes and practices

The search results are presented in a flow diagram following the Preferred Reporting Items for Systematic Reviews and Meta-analyses extension for scoping review (PRISMA-ScR) in Fig. 1 [56].

The characteristics and key findings of the 21 sources of evidence included in this scoping review are presented in Table 1. All 21 papers were published between 2011 and 2023. The majority (90.5%, n = 19) were published within the last decade (2014–2024), with 42.9%, (n = 9) published in the last 5 years (2020–2024). Three out of the six grey literature articles identified were dissertations.

**Table 1:** Summary of the included studies

Author, year	Country	Study design	Study population, sample size	Intervention (if applicable)	Outcome measures	Key findings following study completion (for cross-sectional studies) or at baseline (for interventional studies)	Key findings at the end line (for interventional studies)
Igwe et al., 2011	Nigeria	Cross-sectional study	Pediatric nurses (n = 40) and psychiatric nurses (n = 40)	Not applicable	Knowledge about childhood autism among health workers (KCAHW) questionnaire	The KCAHW mean score was 12.56 ± 3.23 out of a score of 19 The psychiatric nurses had a higher mean score (13.35 ± 2.58) than the pediatric nurses (11.78 ± 3.64)	Not applicable
Namuli et al., 2020	Uganda	Cross-sectional study	Health professionals (n = 36)	Not applicable	Knowledge using the KCAHW questionnaire	The KCAHW scores ranged from 3 to 16 with a median of 12 and a mean of 11.83 (SD = 3.75). 36.1% of the participants had a KCAHW mean score of less than 11.83 Psychiatry resident doctors, clinical psychologists, psychiatrists, and pediatric nurses had KCAHW scores above the study mean score of 11.83. Medical social workers, psychiatric nurses, clinical officers, and pediatricians scored below the study mean score of 11.83	Not applicable
Eseigbe et al., 2015	Nigeria	Cross-sectional study	Medical doctors (n = 167)	Not applicable	Knowledge using the KCAHW questionnaire	The mean KCAHW scores of the participants were 13.5 ± 3.7 and the median 15 Of the participants, 34.7% had seen a case of autism out of whom 34.5% were general practitioners and 65.5% were specialist medical doctors	Not applicable
Sampson et al., 2018	Ghana	Cross-sectional study	Pediatric and psychiatric nurses (n = 223)	Not applicable	Knowledge using the KCAHW questionnaire	The total mean score on the KCAHW questionnaire was 11.74 ± 2.82 for both pediatric and psychiatric nurses out of a possible total mean score of 19 The pediatric nurses’ total mean score was 11.37 ± 2.29 whilst that of psychiatric nurses was 12.11 ± 3.35	Not applicable
Onileimo et al., 2021	Nigeria	A two-group pretest–posttest study design	Pediatric nurses (n = 156)	Intervention: child mental health training is based on the World Health Organization’s mhGAP training manual Comparison: participants who did not receive any training on child mental health	Knowledge and attitude toward child and adolescent mental disorders 38 item scales A 22-item knowledge and attitude questionnaire based on the World Health Organization’s mhGAP behavioral disorder module was added	The knowledge of child mental health disorders mean scores were 89.63 for the intervention group and 85.68 for the control group, out of a maximum score of 96 Attitude mean scores were 10.38 for the intervention groups and 10.55 for the control group, out of a maximum score of 34	The knowledge of child mental health disorders mean scores were 95.36 (intervention groups) and 86.14 (control group) out of a maximum score of 96 Attitude mean scores were 11.01 (intervention groups) and 11.11 (control group) out of a maximum score of 34
Tilahun et al., 2017	Ethiopia	Mixed methods—cross-sectional survey and qualitative study	HEAT—trained health extension workers (HAWs) (n = 104)	HEAT training	Self-reported knowledge, skills, attitude, confidence/competence	Baseline results were not reported	46.1% of the HEWs reported using the child mental health section once or twice a week; which has led to self-reported improvement in knowledge, skills, and attitude Confidence/competence—poor knowledge and skills were highlighted as barriers to integrating child mental health into PHC Most of the participants reported that they had difficulty detecting, supporting, and preventing child mental health problems
Tilahun et al., 2019	Ethiopia	Cross-sectional study	Health extension workers (HEWs) (n = 309)	Two types of educational interventions were provided: (1) the basic mental health module (HEAT) and (2) the enhanced HEAT mental health module (HEAT+) of the upgrading program	Attitude and belief	Baseline results were not reported	HEAT+ trained (Z = − 6.24, p < 0.001, r = − 0.44) and basic HEAT trained (Z = − 6.14, p < 0.001, r = − 0.42) HEWs were more likely to believe that children with autism can improve their language skills compared with untrained HEWs. Two-thirds of the untrained HEWs thought that children with autism would never or rarely improve their language with the right help Compared to HEWs untrained in mental health, both HEAT and HEAT+ trained HEWs were less likely to endorse negative beliefs relating to children with autism. The HEAT+ trained group in turn endorsed fewer negative beliefs than the HEAT-trained group
Tungchama et al., 2019	Nigeria	Cross-sectional study	Health workers (n = 375)	Not applicable	Stigmatizing attitude scale and knowledge of the child and adolescent mental illness (CAMI)	The health workers had a stigmatizing attitude toward CAMI with up to 42% of the health workers did not think that children and adolescents affected by mental illness should be allowed to play with other children and up to 38% would feel ashamed if people knew a child in their family had a mental illness There was poor knowledge of CAMI with up to 25% of the health workers indicating that evil spells cast on a person could result in CAMI. 13% believed that such illness could result from breaking a taboo or sinning against the gods	Not applicable
Akol et al., 2017	Uganda	Cross-sectional	Clinical officers, nurses, and midwives (n = 33)	5 days of CAMH training based on the mhGAP-IG version 1.0 and the International Association of Child and Adolescent Psychiatry and Allied Professions (IACAPAP)	A CAMH knowledge test based on WHO mhGAP IG was administered on day 1 before training and on day 5 after training to explore knowledge	Overall knowledge mean score was 56.2 (95% CI 51.6, 60.9) out of the maximum of 88 The knowledge mean score for clinical officers was 64.0 (95% CI 57.0, 71.0), and for nurses/midwives it was 52.9 (95% CI 47.3, 58.5). Clinical officers had significantly higher mean test scores than nurses and midwives (p < 0.05)	Knowledge mean scores for clinical officers’ post-training were 76.8 (95% CI 72.4, 81.2), and for nurses/midwives were 62.4 (95% CI 56.9, 68.0) The overall knowledge mean score post-training was 66.8 (95% CI 62.4, 71.4). The overall knowledge gain was 18.8% Improved mean scores from pre-to post-test were 20% for clinical officers and 18% for nurses/midwives
Kutcher et al., 2017	Tanzania	The longitudinal cohort study	Clinical officers, registered nurses, midwives, and other community health providers (HCPs) (n = 61)	HCP training using the Adolescent Depression Education Program developed in Canada to train HCPs. Initial training took 5 days, and refresher training took 4 days. The participants were followed up for one year	Knowledge, confidence, practices, and attitude of HCPs related to depression in young people Outcomes were obtained using a questionnaire: pre-training, post-training, pre-refresher training, post-refresher, and post-refresher follow-up	The mean knowledge score was 13.84, SD ± 3.55, out of a maximum score of 30 The mean confidence score was 10.28, SD ± 2.96, out of the maximum score of 16 Baseline data on attitude and practices were not reported	The mean knowledge score post-training was 20.14, SD ± 2.70, pre-refresher training was 19.90, SD ± 2.80 and post-refresher training was 20.12, SD ± 2.53 out of the maximum score of 30 The mean confidence score post-training was 13.14, SD ± 2.33, pre-refresher training was 12.31, SD ± 2.54, and post-refresher was 12.22, SD ± 2.10, out of the maximum score of 16 All participants’ attitudes toward people with mental illnesses or mental health problems had improved Practices: the number of patients being identified, diagnosed, or treated for depression increased
Oshodi et al., 2013	Nigeria	Cross-sectional study, descriptive study	Physicians and nurses in pediatric-related specialties (n = 133)	Not applicable	A questionnaire exploring the health providers’ exposure to CAMH in routine care, their perceived knowledge and competence dealing with CAMH, and perceived areas of need for CAMH training	41.9% of participants reported feeling incompetent in providing care related to CAMH issues, and only 34.4% were aware of any CAMH services in their institution 84.4% of participants highlighted a knowledge gap in CAMH assessment	Not applicable
Sadoo et al., 2022	Uganda	Mixed methods	Healthcare workers who had regular contact with children under 2 years from health centers (n = 93)	Baby Ubuntu early intervention program A structured early child development training program	Pre- and post-training impact on knowledge, attitude, practice, and confidence before and after the initial training session, and immediately before the refresher training 6 months later	The knowledge median score was 4.0 (IQR 3–5) out of the maximum 8 and the confidence median score was 2.7 (IQR 2–4) out of the maximum 5	The knowledge median score was 7.0 and the confidence median score was 4.7. The increase in post-training for both was significant with a p-value of < 0.001 Before refresher training, the knowledge and confidence score median dropped to 6.0 and 4.0, respectively, but remained above the baseline levels The HCWs self-reported increased practices and improved attitudes toward children with disabilities due to the training. They have a better understanding of how to assess, manage, and refer to children with disabilities
Zeleke et al., 2018	Ethiopia	Cross-sectional study	Caseworkers, teachers, nurses, counselors, psychologists, therapists, social workers, special needs educators, and therapeutic care workers (n = 80)	Not applicable	Survey questionnaire to explore participants’ confidence in their understanding of autism	66.8% of the participants felt that their preservice education did not adequately prepare them to address autism The respondents were most confident in understanding autism, where overall they reported being 48% to 88% confident to very confident Only 1% of respondents reported not being confident in understanding autism	Not applicable
Akinyemi et al., 2017	Nigeria	Cross-sectional	191 Nurses and community health workers (CHWs) (n = 371)	Not applicable	The self-administered semi-structured questionnaire, namely 1. The awareness of child and adolescent mental health inventory 2. Healthcare providers’ practices concerning child and adolescent inventory	The nurses and CHWs had little knowledge of CAMH disorders. Nurses were more knowledgeable about CAMH than CHWs Good knowledge about CAMH among the respondents did not translate to good practice regarding CAMH services. 73.5% of the participants reported limited confidence in diagnosing a child or adolescent with mental illness	Not applicable
Sheriff et al., 2022	Ghana	Exploratory, qualitative study	Nurses, pediatricians, general medical officers, physician assistants, and midwives (n = 37)	Not applicable	A face-to-face, 10–15 min free-listing interviews	More than a third of the participants incorrectly listed epilepsy, infectious diseases, and/or nutritional disorders as developmental disorders (DDs) DDs with physical symptoms were the most salient among healthcare workers. DDs such as learning disabilities were overlooked. ADHD and intellectual disabilities were the least salient listed DDs Only 23% of the participants could list one cause of cerebral palsy	Not applicable
Rodin et al., 2021	Uganda	Mixed Methods	Healthcare professionals (n = 152)	Not applicable	Survey and semi-structured interviews to explore the professional experiences, knowledge, and perceptions of healthcare workers on Tic disorders and Tourette syndrome	48.7% of the respondents did not feel confident in making a diagnosis of tic disorder and only 36.6% were confident 75.9% highlighted that lack of knowledge about tic disorders was the most prevalent challenge identified by health professionals This paper highlights the need for all healthcare professionals to receive adequate training regarding tic disorders	Not applicable
Muke et al., 2023	Congo	Cross-sectional study	Health professionals in pediatrics—general practitioners, nurses, nutritionists, and training students (n = 26)	Not applicable	A survey form on theoretical knowledge, attitude, and practices on psychomotor development and psychomotor disorders	69.2% of the participants were reported to lack theoretical knowledge about child psychomotor development resulting in late diagnosis and consequently poor treatment of psychomotor development disorders	Not applicable
Tasew et al., 2021	Ethiopia	Cross-sectional study	Nurses (n = 331)	Not applicable	Knowledge of autism using the KCAHW questionnaire	The KCAHW mean score was 8.79 ± 0.44 Social interactions domain had a mean score of 3.75 ± 0.23. 53.47% of the nurses scored at and above the mean score Impairment in the communication domain had a mean score of 0.535 ± 0.05 53.5% of the nurses scored at and above the mean score Knowledge about obsessive and repetitive characteristics mean—1.73 ± 0.13. 56.2% of the nurses scored at and above the mean score Domain on comorbid conditions, autism curability, and its onset—2.8 ± 0.17. 54.8% of the nurses scored at and above the mean score	Not applicable
Williams et al., 2018	South Africa	Cross-sectional study	Nurses in primary health care (n = 275)	Not applicable	Knowledge about childhood autism among health workers (KCAHW) questionnaire Perception of autism spectrum disorder questionnaire	The KCAHW questionnaire mean score was 10.52. The obsession and compulsive behavior in autistic children were the least understood domain with only 58.2% of the participants scoring above the mean score (2.33). Standard deviation not reported Most of the nurses did not have negative perceptions and beliefs about the causes of autism 88% of the participants agreed that they would benefit from further training on the identification and diagnosis of autism spectrum disorder	Not applicable
Matlou et al., 2021	South Africa	Cross-sectional study	Health care practitioners in the pediatric department (n = 116)	Not applicable	An in-depth questionnaire focusing on screening and surveillance; diagnosis and tools used; and planned management	The health workers were not well informed about the diagnosis of autism and lacked confidence in assessment Autism is not screened for routinely or as recommended in practice	Not applicable
Fatma et al., 2021	Kenya	Cross-sectional study	Health workers—nurses, medical officers, clinical officers, consultant pediatrics, residents (pediatrics, psychiatry, pediatric surgery) (n = 230)	Not applicable	Knowledge of autism using the self-administered KCAHW questionnaire Focused group discussions In-depth interviews	The KCAHW questionnaire mean score was 14.4 ± 2.4. (benchmark for good or poor knowledge) 52.2% of the participants had a score above the mean. Good knowledge, 48.8% of the participants had a score below the mean—poor knowledge of autism Better knowledge among the health workers in specialties (pediatrics, psychiatry, pediatric surgery) The highest knowledge gap was reported in the social interactions’ domain and impairment in the communication domain	Not applicable

### Study designs

Of the 21 sources of evidence included in the scoping review, 15 were cross-sectional studies [28, 33, 35, 39–41, 43, 57–64], one was a longitudinal cohort study [65], one was an experimental pretest–posttest design [27], two were mixed methods papers [22, 66], and two were exploratory studies [67, 68].

### CAMH disorders

The most described CAMH disorder was autism, which was reported in ten papers [33, 39, 40, 57, 58, 60, 62–64, 68], papers described CAMH in general [27, 28, 35, 43], one reported on child and adolescent mental illness [41], two reported on developmental disorders [22, 67], one on child mental health [59], one on adolescent depression [65], one on Tourette’s Syndrome [66], and one on psychomotor development of children [61].

### Geographic distribution

**Fig. 2:**
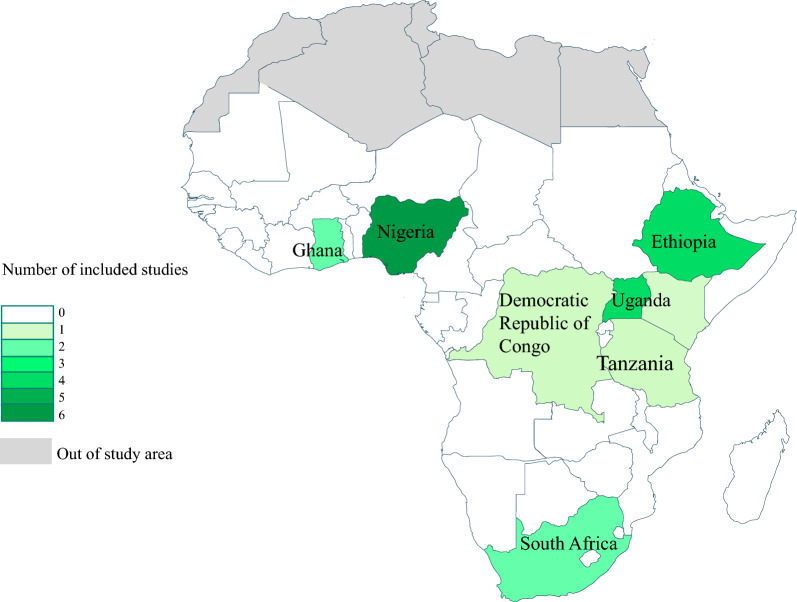
Geographical distribution of SSA countries in included studies

There were six studies from Nigeria [27, 35, 39, 41, 43, 57], and four from Uganda [22, 28, 40, 66]. Four studies were from Ethiopia [33, 59, 68], two from Ghana [58, 67], two from South Africa [60, 63], one from Congo [61], one from Kenya [64], and one from Tanzania [65]. The geographic distribution of studies is shown in Fig. 2.

### Target population

The healthcare workers represented in the reviewed studies were mostly nurses [22, 27, 28, 35, 41, 43, 57, 58, 61–65, 67, 68], followed by midwives [22, 28, 65, 67] and medical doctors [39, 41, 43, 64, 67]. Clinical officers were evaluated in three studies [22, 28, 64, 65]. Other health professionals included in the studies were health extension workers [33, 59] and community HCWs [35, 65]. Two of the studies focused on psychologists and social workers [41, 68]. Some papers used the general terminology ‘healthcare workers’ or ‘health professionals’ or ‘health care practitioners [40, 60, 66].

### Measures of evaluation

Most of the studies (57.1%) did not use a standardized tool to evaluate the outcomes of interest [22, 33, 35, 43, 59, 62–68]. One-third (33.3%) of the studies used the Knowledge about Childhood Autism among HCWs (KCAHW) questionnaire [39, 40, 57, 58, 62–64]. The two studies that trained HCWs using mhGAP guidelines utilized the standard mhGAP knowledge test [27, 28]. One study conducted in Nigeria used a Stigmatizing Scale and a Knowledge of Child and Adolescent Mental Illness Scale [41].

### Synthesis of results on knowledge, attitudes, and practices

Ten of the included studies reported on knowledge only [39, 40, 57, 58, 61–64, 67, 68]. Only two papers evaluated all three outcomes of interest related to the review, namely, knowledge, attitude, and practices related to CAMH [61, 65]. One paper reported on attitudes [33], one on both knowledge and practices [35], and two studies on knowledge and attitudes toward CAMH [28, 41]. Several studies that reported knowledge, attitudes, or practices also reported other related concepts, such as confidence, beliefs, and competence [22, 33, 35, 43, 59, 60, 69]. The sections below summarize the findings related to HCWs’ CAMH knowledge, attitudes, and practices for the studies included in this review.

### Knowledge related to CAMH

Seven studies (two in Nigeria and one in Ghana, Ethiopia, Kenya, South Africa, and Uganda) that used the KCAHW questionnaire showed an evident knowledge gap about childhood autism among healthcare providers [39, 40, 57, 58, 62–64]. The KCAHW questionnaire has 19 items and a maximum score of 19. It is grouped into four domains: (i) social interaction; (ii) communication; (iii) circumscribed and repetitive behaviors; and (iv) autism characteristics and comorbidities [70]. A score above the mean of the KCAHW scores was considered good knowledge of autism. Nurses in Ethiopia had the lowest KCAHW mean score (8.79 ± 0.44) [62], while the highest score was reported in Kenya (14.4 ± 2.4) [64]. In Nigeria, all participants who had experience nursing children with autism achieved scores of 15 or above and psychiatric nurses outperformed pediatric nurses in all four domains of the questionnaire [57]. In Uganda, 36.1% of the participants scored below the mean (11.8 ± 3.8) [40].

Three studies in Uganda highlight the lack of knowledge among healthcare workers regarding child and adolescent mental health (CAMH) and developmental disabilities, with a significant knowledge gap regarding Tourette Syndrome [22, 28, 66]. In Nigeria, a notable lack of knowledge of child and adolescent mental illnesses was reported. Approximately 25% of the HCWs believed that child and adolescent mental illnesses could be caused by evil spells cast on a person [41], and 13% thought that such an illness could result from breaking a taboo or sinning against the gods [41].

### Attitudes toward CAMH

Eight studies evaluated the attitudes of healthcare workers (HCWs) toward child and adolescent mental health (CAMH) in different countries [22, 27, 33, 41, 59, 61, 63, 65]. The findings indicate that HCWs in Ethiopia had slightly positive attitudes, but still harbored negative beliefs about children with autism [33, 59]. Health extension workers expressed doubts about the children’s ability to make their parents proud, attend school, get married in the future, and play normally with other children [33]. The other study highlighted that health extension workers’ negative attitudes and stigma act as barriers to the integration of child mental health services into primary care [59]. In Nigeria, HCWs showed poor attitudes toward CAMH, with a significant percentage believing that children with mental illness should not be allowed to play with other children [27, 41]. Up to 38% said that they would feel ashamed if someone knew that a child in their family had mental illness [41]. However, in South Africa, most of the nurses did not have negative perceptions and beliefs about the causes of autism [63].

### Practices toward CAMH

Three studies indicated that HCWs’ practices toward CAMH were poor [22, 43, 68]. In Nigeria, participants expressed feelings of incompetence in managing CAMH problems [43]. Similarly, in Ethiopia, most of the participants believed that their pre-service education did not adequately prepare them to work with children with autism [68]. As a result, 66.8% of the participants felt ill-equipped to address autism and, consequently, did not provide services to children with autism [68]. Additionally, a study conducted in Uganda highlighted a significant gap in practices among HCWs concerning children with developmental disabilities, with only 148 appropriate referrals made annually, averaging 12.3 per month [22].

### Other findings related to KAP on CAMH

Five studies reported that HCWs felt incompetent and lacked the confidence to deliver CAMH services [22, 35, 59, 60, 65]. In Tanzania, the participants reported changes in anxiety levels after training on adolescent depression, with 48% feeling more anxious and 43% feeling less anxious and more confident [65]. In South Africa, the health workers lacked confidence in assessment and therefore, autism is not screened for routinely or as recommended in practice [60].

### Targeted interventions for CAMH

Fifteen of the 21 sources of evidence included in the review were non-interventional studies [35, 39–41, 43, 57, 58, 60–64, 66–68]. Six of the eligible these studies trained health professionals on CAMH and evaluated the impact of the training [22, 27, 28, 33, 59, 65]. These studies included innovative programs such as the Health Education and Training—“Mental Health: Resources for Community Health Workers” program which comprises five training videos focusing on childhood developmental problems and WHO mhGAP guidelines as well as a certified adolescent depression education program, and the Baby Ubuntu program [22, 27, 28, 33, 59, 65, 71]. The studies reported an overall increase in knowledge among the healthcare workers who participated in the training programs. Details of the training program is included in Table 1.

## Discussion

Healthcare workers’ adequate knowledge, positive attitudes, and effective practices in CAMH can promote early identification and management of CAMH problems while reducing the stigma associated with seeking CAMH care services [22, 26–28, 43]. This scoping review mapped out the extent of literature on knowledge, attitudes, and practices toward CAMH among HCWs in SSA.

A total of 21 studies were identified, with approximately two-thirds of the studies from three countries in SSA (Uganda, Ethiopia, and Nigeria) [22, 27, 28, 33, 35, 39–41, 43, 57, 59, 62, 65, 66]. The findings highlight the evidence gap in the literature regarding knowledge, attitudes, and practices toward CAMH among HCWs in SSA. The lack of literature from other parts of SSA may be due to a scarcity of expertise and resources for research on this topic [72]. Increasing research efforts in SSA countries would help provide a more comprehensive understanding of CAMH [20, 69, 73].

The papers included in the review had a diverse study population. Medical doctors, nurses, clinical officers, midwives, psychologists, social workers, and community health workers were among the study participants. The diversity of the HCWs engaging in CAMH research brings together a wide range of skills, experiences, and perspectives, leading to a more integrated and effective approach to addressing CAMH challenges [2, 74]. Collaboration of the different cadres of HCWs in addressing CAMH issues has been emphasized, as it can facilitate access to child mental health services [28, 75, 76].

The most studied CAMH disorder was autism, which was reported in nearly half of the sources of evidence from Ethiopia, Uganda, and Nigeria [33, 39, 40, 57, 58, 68]. This could be due to the high prevalence of autism, which affects approximately 1 in 100 children worldwide, and the complexity of its presentation [77, 78]. Autism has a wide range of symptoms, levels of severity, and is often comorbid with other conditions. Insufficient diagnostic capacity and management of autism increases the need for more research to help address these challenges [79–81]. It is possible that the growing awareness and advocacy for autism, as well as increased funding from charitable organizations, have contributed to its status as one of the most studied CAMH disorders [82, 83].

A significant gap in knowledge of CAMH among healthcare workers was reported in most studies. Within studies, variations were observed in knowledge levels related to CAMH among cadres of healthcare workers [28, 40, 57, 58, 64]. Knowledge about autism was a prominent theme in the reviewed studies, with notable knowledge gaps reported. Studies conducted in Nigeria, Ghana, South Africa, Kenya, and Uganda using the KCAHW questionnaire show varied mean scores from 8.79 ± 0.44 to 14.4 ± 2.4, indicating differences in understanding of autism among healthcare workers [39, 40, 57, 58, 62–64]. The KCAHW scores were comparable with scores reported in similar studies conducted in Türkiye, Saudi Arabia, and Italy, with lowest scores observed in China (7.3 ± 2.19), while a study done in Sri Lanka reported a mean of 13.23 ± 2.65 [84–89]. Access to specialized training, supervision opportunities, sufficient educational curricula, and allocation of resources for capacity building in CAMH for healthcare workers varies in SSA countries. This could explain the differences in knowledge levels regarding autism among different countries in the region [18, 28, 76, 90]. Therefore, increasing awareness and education on CAMH, as well as providing targeted training programs tailored to the specific needs of different healthcare professions in the area, is necessary.

Overall, most studies reported that HCWs had poor attitudes toward CAMH [22, 27, 33, 41, 59, 65]. This attitude is not only prevalent in low-income countries but also in high-income countries such as the United Kingdom and Slovenia [91, 92]. The poor attitudes could be attributed to HCWs’ lack of knowledge about CAMH, which can lead to misconceptions and stigma toward CAMH [79]. Cultural norms and beliefs may stigmatize CAMH, leading to reluctance to seek or provide appropriate CAMH care [34, 93]. These negative beliefs and misconceptions about the causes and treatment of mental health disorders contribute to the stigma, which impedes early detection and intervention efforts [41]. However, some studies have reported positive perceptions of CAMH by healthcare workers in certain regions, for example, in South Africa, majority of the nurses did not have negative perceptions and beliefs about the causes of autism [58]. Given the complexity of changing attitudes, a more comprehensive approach involving community engagement and awareness campaigns to address cultural beliefs and reduce stigma may be needed [94, 95]. Additionally, fostering supportive work environments for HCWs and policy-level interventions related to CAMH is crucial [96–98]. By doing so, we can help diminish the stigma surrounding CAMH and enhance early detection and intervention efforts.

Practices toward CAMH were explored in a limited number of studies [22, 43, 59, 68, 69]. The studies have revealed a need for improvement, particularly in Nigeria and Ethiopia, where healthcare professionals were reported to have poor practices and feel ill-prepared to address CAMH disorders [43, 59]. Additionally, studies conducted in Tanzania, Ethiopia, Nigeria, and Uganda revealed that HCWs lacked confidence and competence in delivering CAMH services [22, 35, 59, 69]. Similarly, in India, doctors reported feeling unconfident in managing childhood psychiatric illnesses [99]. The scarcity of providers with specific competencies in CAMH is a global concern [100], due to the limited focus on CAMH in pre-service education programs and the lack of ongoing professional development opportunities [2, 18]. To promote CAMH services and enhance practices and competence, comprehensive training programs that address assessment, management, and referral processes are necessary [24, 75, 101, 102]. Targeted educational programs can also enhance self-confidence and competence in addressing CAMH issues [22, 103].

It is worth noting that six of the studies included reported a positive impact on HCWs’ knowledge, attitudes, and practices regarding post-CAMH-related training interventions [22, 27, 28, 33, 59, 65]. Therefore, targeted training, for example, using the mhGAP guidelines, the HEAT training, Baby Ubuntu, can effectively enhance HCWs’ understanding of CAMH-related issues and improve their attitudes and practices regarding CAMH [22, 27, 28, 59, 60, 65] and, in the future, bridge the CAMH services gap in SSA. Designing training programs on child and adolescent mental health requires careful consideration of cultural and socio-contextual factors [104]. It is crucial to acknowledge and respect cultural diversity, language barriers, and family dynamics [105]. Additionally, socioeconomic status, stigma, and discrimination can impact mental health outcomes and access to services [106]. By addressing these factors, training programs can equip the HCWs with KAP to meet the diverse needs of children, adolescents, and their families on matters CAMH in SSA.

## Strengths and limitations

An extensive search was conducted, enabling the identification of a considerable number of studies. The scoping review methodology included various study designs and used a systematic approach to identifying relevant studies, charting them, and analyzing the selected study outcomes. While not required for scoping reviews, an assessment of the quality and risk of bias in the studies included enhanced the robustness of the review. The first limitation we would like to acknowledge is relying solely on the KAP framework may lead to overlooking significant aspects of mental health literacy. In contrast, frameworks like the Mental Health Literacy framework not only focus on knowledge and attitudes but also on the abilities and skills required to identify, manage, and prevent mental illness at individual and community level [46]. This approach offers a more comprehensive and results-oriented approach to mental health. Second, there are no universal cut-off scores for the KCAHW scale, a tool used to measure knowledge of autism. As a result, we relied on the guidelines provided by each author to determine the cut-off scores. For example, some studies used mean scores to categorize knowledge levels as poor or good, while others used quartiles, and this could have limited the synthesis of the included articles that used KCAHW scale.

## Conclusion

In conclusion, this scoping review demonstrates the need for evidence-based targeted interventions and approaches to improve knowledge, attitudes, and practices related to CAMH among HCWs in SSA. Examples include training programs, improving educational curricula, providing ongoing training opportunities, implementing community-wide awareness campaigns, and addressing contextual factors in promoting CAMH services. By bridging the evidence gap and enhancing the capacity of HCWs, it is possible to improve CAMH outcomes and reduce the CAMH service gap in the region.

### Supplementary Information

Supplementary Material 1.

Supplementary Material 2.

## Data Availability

Not applicable.

## Abbreviations

CAMH: Child and Adolescent Mental Health
HCWs: Healthcare workers
KAP: Knowledge, attitudes, and practices
KCAHW: Knowledge about Childhood Autism among Health Workers
mhGAP: Mental Health Gap Action Programme
SSA: Sub-Saharan Africa
WHO: World Health Organization
