# Supplementary material for: Knowledge, attitudes, and practices on child and adolescent mental health among healthcare workers in sub-Saharan Africa: a scoping review
Source: Int J Ment Health Syst. 2024 Jul 16;18:27. doi: 10.1186/s13033-024-00644-8 (PMC11253363; doi:10.1186/s13033-024-00644-8)
Supplement: Supplementary file 1 — Supplementary Material 1. [file 13033_2024_644_MOESM1_ESM.docx]

**Appendix 1: List of sub-Saharan countries included in the search term**

The SSA countries included are Angola, Benin, Botswana, Burkina Faso, Burundi, Cameroon, Cape Verde, Central African Republic, Chad, Comoros, Republic of the Congo, Democratic Republic of the Congo, Cote d'Ivoire, Djibouti, Equatorial Guinea, Eritrea, Ethiopia, Gabon, Gambia, Ghana, Guinea, Guinea-Bissau, Kenya, Liberia, Madagascar, Malawi, Mali, Mauritania, Mauritius, Mozambique, Namibia, Niger, Nigeria, Rwanda, Sao Tome and Principe, Senegal, Seychelles, Sierra Leone, Somalia, South Africa, South Sudan, Sudan, Swaziland, Tanzania, Togo, Uganda, Zambia, and Zimbabwe.

**Appendix 2: Search strategy -PubMed**

| **Search query** | **Search topic** | **Search keywords (titles, abstracts, general keywords, and subject headings)** |
| --- | --- | --- |
| 1 | Population | nurse*[MeSH Terms] OR community HCWs[Mesh] OR health personnel[MeSH Terms] OR HCW*[tiab] OR HCW*[tiab] OR HCW[tiab] OR doctor*[tiab] OR clinician*[tiab] OR health professional*[tiab] OR health workforce*[tiab] OR primary health care work*[tiab] OR primary healthcare work*[tiab] OR primary healthcare profession*[tiab] OR primary care team*[tiab] OR community health work*[tiab] OR health extension work*[tiab] OR psychiatrist*[tiab] OR psychologist*[tiab] OR social worker*[tiab] OR nursing staff, hospital[Mesh] OR occupational therap*[tiab] OR counselor*[tiab] OR conselor*[tiab] OR clinical officer*[tiab] OR psychosocial work*[tiab] |
| 2 | Concept 1 | autism spectrum disorder[MeSH Terms] OR autistic disorder[Mesh] OR autism*[tiab] OR ADHD[tiab] OR attention deficit disorder with hyperactivity[MeSH Terms] OR attention deficit hyperactivity disorder[tiab] OR intellectual disability[MeSH Terms] OR communication disorders[Mesh] OR neurodevelopmental disorders[MeSH Terms] OR anxiety disorders[MeSH Terms] OR trauma and stressor related disorders[MeSH Terms] OR depressive disorder[Mesh] OR depression[MeSH Terms] OR child mental health[All Fields] OR adolescent mental health[All Fields] OR child and adolescent mental health[All Fields] OR CAMH*[All Fields] OR CAMI[All Fields] OR NDD[tiab] OR DD[tiab] OR ID[tiab] OR conduct disorder[tiab] OR learning disabilit*[tiab] OR specific learning disorder[tiab] OR specific learning difficult*[tiab] OR dyslexia OR conduct problem* OR fragile X[tiab] OR panic[Mesh] OR mutism[Mesh] OR anxiety[tiab] OR panic[tiab] OR phobic[tiab] OR phobia[tiab] OR stress*[tiab] OR posttraumatic[tiab] OR posttraumatic[tiab] OR traumatic stress[tiab] OR psychological trauma*[tiab] OR depress*[tiab] OR selective mutism*[tiab] OR elective mutism*[tiab] OR child and adolescent mental health problem*[tiab] OR child and adolescent mental health disorder*[tiab] OR adolescent psychiatry[Mesh] OR child psychiatry[Mesh] OR language development disorders[Mesh] OR developmental disabilities[Mesh] OR learning disabilities[Mesh] OR problem behavior[Mesh] OR problem behavior[tiab] OR child development disorders, pervasive[Mesh] OR developmental disab*[tiab] OR developmental disord*[tiab] OR developmental delay*[tw] |
| 3 | Concept 2 | health knowledge, attitudes, practice[MeSH Terms] OR knowledge, attitude and practice[tiab] OR attitude[MeSH Terms] OR knowledge[MeSH Terms] OR practice[tiab] OR KAP[tiab] OR attitude of health personnel*[Mesh] OR capacity build*[tiab] OR capacity strength*[tiab] OR skill*[tiab] OR behavior*[tiab] OR behavior chang*[tiab] OR behavior*[tiab] OR behavior chang*[tiab] OR competenc*[tiab] OR knowledge[tiab] OR attitud*[tiab] OR practic*[tiab] OR practis*[tiab] OR empower*[tiab] OR belief[tiab] OR clinical competence[Mesh] OR health literacy[Mesh] |
| 4 | Context | "Africa South of the Sahara" OR "Sub Saharan Africa"[tiab] OR "sub-Saharan Africa"[tiab] OR "SSA" OR "Sub-Saharan Africa"[tiab] OR "Angola"[tiab] OR "Benin"[tiab] OR "Botswana"[tiab] OR "Burkina Faso"[tiab] OR "Upper Volta"[tiab] OR "Burundi"[tiab] OR "Cameroon"[tiab] OR "Cape Verde"[tiab] OR "Central African Republic"[tiab] OR "Chad"[tiab] OR "Comoros" [tiab] OR "Congo"[tiab] OR "Cote D’ivoire"[tiab] OR "Ivory Coast"[tiab] OR "Zaire"[tiab] OR "Democratic Republic Of The Congo"[tiab] OR " French Somaliland"[tiab] OR "Djibouti"[tiab] OR "Equatorial Guinea"[tiab] OR "Eritrea"[tiab] OR "Ethiopia"[tiab] OR "Gabonese Republic"[tiab] OR "Gabon"[tiab] OR "Gambia"[tiab] OR "Gold Coast"[tiab] OR "Ghana"[tiab] OR "Guinea"[tiab] OR "Guinea-Bissau"[tiab] OR "Kenya"[tiab] OR "Basutoland"[tiab] OR "Lesotho"[tiab] OR "Liberia"[tiab] OR "Malagasy Republic"[tiab] OR "Madagascar"[tiab] OR "Nyasaland"[tiab] OR "Malawi"[tiab] OR "Mali"[tiab] OR "Mauritania"[tiab] OR "Mauritius"[tiab] OR "Mayotte"[tiab] OR "Mozambique"[tiab] OR "Namibia"[tiab] OR "Niger"[tiab] OR "Nigeria"[tiab] OR "Reunion"[tiab] OR "Rwanda"[tiab] OR "Ruanda-Urundi"[tiab] OR "Sao Tome & Principe"[tiab] OR "Sao Tome"[tiab] OR "Senegal"[tiab] OR "Seychelles"[tiab] OR "Sierra Leone"[tiab] OR "Somalia"[tiab] OR "South Africa"[tiab] OR "South Sudan"[tiab] OR "Sudan"[tiab] OR "Swaziland"[tiab] OR "Eswatini"[tiab] OR "Togolese Republic"[tiab] OR "Togo"[tiab] OR "Uganda"[tiab] OR "United Republic Of Tanzania"[tiab] OR "Tanzania"[tiab] OR "Zambia"[tiab] OR "Zimbabwe"[tiab] OR "Rhodesia"[tiab] OR "Africa Eastern"[tiab] OR "Africa Southern"[tiab] OR "AfricaWestern"[tiab] OR "Africa Central" [tiab] |
| 5 | Combined search query | #1 AND #2 AND #3 AND #4 |

**Appendix 3: Data extraction template**

| ***Author, Year*** | ***Country*** | ***Study***  ***Design***  ***/Setting/***  ***Sample***  ***Size*** | ***Study***  ***Aims*** | ***Study***  ***Population*** | ***Intervention***  ***(What,***  ***Who,***  ***Duration)*** | ***Comparator*** | ***Outcome Measures*** | ***Key Findings related to the Review*** | ***Limitations*** | ***Other Comments*** |
| --- | --- | --- | --- | --- | --- | --- | --- | --- | --- | --- |
|  |  |  |  |  |  |  |  |  |  |  |
|  |  |  |  |  |  |  |  |  |  |  |
|  |  |  |  |  |  |  |  |  |  |  |
