# Supplementary material for: Knowledge, attitudes, and practices on child and adolescent mental health among healthcare workers in sub-Saharan Africa: a scoping review
Source: Int J Ment Health Syst. 2024 Jul 16;18:27. doi: 10.1186/s13033-024-00644-8 (PMC11253363; doi:10.1186/s13033-024-00644-8)
Supplement: Supplementary file 2 — Supplementary Material 2. [file 13033_2024_644_MOESM2_ESM.docx]

Table 1: Quality scores of the included cross-sectional studies based on the Newcastle-Ottawa quality assessment tool.

| **First author (publication year)** | **Selection of participants** | | | | **Comparability** | **Ascertainment of Outcome** | | Total score | **Quality grade** |
| --- | --- | --- | --- | --- | --- | --- | --- | --- | --- |
|  | **Sample representativeness** | **Sample size** | **Non-respondents** | **Exposure ascertainment** | **Comparability of subjects in outcome groups** | **Outcome assessment** | **Statistical test appropriateness** |  |  |
| Igwe et al., 2011 | ***** | ***** | ***** | ****** | **-** | ***** | ***** | 7/10 | **Good** |
| Namuli et al., 2020 | ***** | ***** | ***** | ****** | ***** | ***** | ***** | 8/10 | **Good** |
| Eseigbe et al., 2015 | **-** | ***** | **-** | ****** | ***** | ***** | ***** | 6/10 | **Satisfactory** |
| Sampson et al., 2018 | **-** | ***** | **-** | ****** | ***** | ***** | ***** | 6/10 | **Satisfactory** |
| Tungchama et al., 2019 | ***** | ***** | ***** | ***** | ***** | ***** | ***** | 7/10 | **Good** |
| Tilahun et al., 2017 | **-** | ***** | ***** | ***** | ***** | ***** | ***** | 6/10 | **Satisfactory** |
| Tilahun et al., 2019 | **-** | ***** | ***** | ***** | ***** | ***** | ***** | 6/10 | **Satisfactory** |
| Oshodi et al., 2013 | **-** | **-** | ***** | ***** | **-** | ***** | **-** | 3/10 | **Unsatisfactory** |
| Zeleke et al., 2018 | ***** | ***** | ***** | ***** | **-** | ***** | ***** | 6/10 | **Satisfactory** |
| Akinyemi et al., 2017 | ***** | ***** | ***** | ***** | ****** | ***** | ***** | 8/10 | **Good** |
| Sheriff et al., 2022 | ***** | ***** | **-** | ***** | ****** | ***** | ***** | 7/10 | **Good** |
| Rodin et al.,2021 | **-** | **-** | **-** | ***** | ***** | ***** | ***** | 4/10 | **Unsatisfactory** |
| Muke et al.,2023 | **-** | **-** | **-** | ***** | ***** | ***** | ***** | 4/10 | **Unsatisfactory** |
| Tasew et al.,2021 | ***** | ***** | ***** | ****** | ***** | ***** | ***** | 8/10 | **Good** |
| Matlou MJ et al.,2021 | ***** | **-** | **-** | **-** | **-** | ***** | ***** | 3/10 | **Unsatisfactory** |
| Williams NA. et al.,2018 | ***** | ***** | ***** | ****** | ***** | ***** | ***** | 8/10 | **Good** |
| Fatma ZSK et al.,2021 | ***** | ***** | **-** | ****** | ***** | ***** | ***** | 7/10 | **Good** |

Table 2: Quality scores of the included cohort study based on the Newcastle-Ottawa quality assessment tool.

| **First author (publication year)** | **Selection of participants** | | | | **Comparability** | **Outcome** | | | **Total score** | **Quality grade** |
| --- | --- | --- | --- | --- | --- | --- | --- | --- | --- | --- |
|  | **Representativeness of exposed cohort** | **Selection of non-exposed cohort** | **Ascertainment of exposure** | **Demonstration that outcome of interest was not present at start of study** | **Comparability of cohorts on the basis of design or analysis** | **Assessment of outcome** | **Length of follow-up** | **Adequacy of follow-up of cohorts** |  |  |
| Onileimo et al., 2021 | ***** | ***** | ***** | ***** | ***** | **-** | **-** | ***** | 6/10 | **Satisfactory** |
| Kutcher et al., 2017 | ***** |  | ***** | ***** | ***** | **-** | ***** | ***** | 6/10 | **Satisfactory** |
| Sadoo et al., 2022 | **-** |  | ***** | ***** | ***** | **-** | ***** | **-** | 4/10 | **Unsatisfactory** |
| Akol et al., 2017 | **-** |  | ***** | ***** | ***** | ***** | **-** | **-** | 4/10 | **Unsatisfactory** |
